# Generation and Characterization of a MYF5 Reporter Human iPS Cell Line Using CRISPR/Cas9 Mediated Homologous Recombination

**DOI:** 10.1038/srep18759

**Published:** 2016-01-05

**Authors:** Jianbo Wu, Samuel D. Hunt, Haipeng Xue, Ying Liu, Radbod Darabi

**Affiliations:** 1 Center for Stem Cell and Regenerative Medicine (CSCRM), Brown Foundation Institute of Molecular Medicine (IMM), University of Texas Health Science Center at Houston, Houston, TX 77030, USA; 2 Department of Neurosurgery, University of Texas Health Science Center at Houston, Houston, TX 77030, USA; 3 The Senator Lloyd & B.A. Bentsen Center for Stroke Research, The Brown Foundation Institute of Molecular Medicine (IMM), University of Texas Health Science Center at Houston, Houston, TX 77030, USA

## Abstract

Human iPS cells hold great promise for disease modeling and treatment of degenerative disorders including muscular dystrophies. Although a few research groups have used them for skeletal muscle differentiation, most were based on gene over-expression or long-term mesenchymal differentiation and retrospective identification of myogenic cells. Therefore, this study was aimed to generate a knock-in reporter human iPS cell line for MYF5, as an early myogenic specification gene, to allow prospective identification and purification of myogenic progenitors from human iPS cells. By using a CRISPR/Cas9 double nickase strategy, a 2A-GFP reporter was inserted before the stop codon of the MYF5 gene using homologous recombination. This approach allowed for highly efficient in-frame targeting of MYF5 in human iPS cells. Furthermore, in order to prove the reporter function, endogenous MYF5 expression was induced using a novel dead Cas9-VP160 transcriptional activator. Induced clones demonstrated appropriate MYF5-GFP co-expression. Finally, to confirm the differentiation potential, reporter human iPS clones were differentiated through embryoid body method and MYF5-GFP^+^ myogenic cells were sorted and characterized. These data provides valuable guidelines for generation of knock-in reporter human iPS cell lines for myogenic genes which can be used for disease modeling, drug screening, gene correction and future *in vivo* applications.

Skeletal muscle is the largest organ in the body with a tremendous regeneration potential. Indeed, its continuous growth and regeneration during life is exceptional, however, it is still prone to many pathologic conditions which might occur at different ages^1^,^2^. Among these, genetic disorders such as muscular dystrophies (MDs), age-related sarcopenia, and muscle cachexia are the most common ones^2^,^3^,^4^. Although the etiologies of these disorders are heterogeneous, the final outcome in all of these is common as they eventually lead to gradual muscle atrophy and its replacement with fibrotic or fat tissue^5^,^6^. Therefore study of these muscle disorders and their treatment is an important health concern.

Fortunately, with the recent advancements of generating induced Pluripotent Stem Cells (iPS cells) from somatic cells, different lineage progenitors can be generated from patient samples which can be used for *in vitro* disease modeling, drug screening, gene correction and finally as a cell based therapy for muscle disorders^7^,^8^,^9^,^10^,^11^. Thus, myogenic differentiation of iPS cells is critical for successful application of iPS cells. However, directed differentiation of human iPS cells toward myogenic lineage is a challenging task due to paucity of paraxial mesoderm progenitors during *in vitro* differentiation of iPS cells.

For this reason, a few research groups including us have started working on human iPS cells to develop strategies for differentiation toward skeletal muscle. A majority of these efforts were based either on transient myogenic genes over-expression (PAX3, PAX7, and MYOD) or differentiation toward mesodermal/mesenchymal lineage^12^,^13^,^14^,^15^,^16^,^17^,^18^,^19^. However, the need for lentiviral over-expression of myogenic genes was the major limiting factor especially if one envisions future possible clinical application of the cells. Although a few other methods have recently been developed to induce myogenesis using Wnt agonists, the purity of the outgrowth were not clear and the readout for myogenic commitment were based on retrospective gene expression and immunostaining on explants^17^,^18^,^19^,^20^.

Therefore, in the current study, we planned to generate a knock-in reporter human iPS cell line for an early myogenic gene (such as MYF5). This will allow us and other scientists to use this approach for directed differentiation of human iPS cells toward myogenic progenitors and to study temporal emergence of myogenic progenitors during differentiation using a prospective strategy.

We chose MYF5 as it is one of the earliest myogenic determination genes in the somite and its unique transcriptional isoform, makes it suitable for our targeting strategy^21^,^22^,^23^. In order to have an accurate reporter activity, we have targeted the last exon of the MYF5 gene using a 2A-GFP reporter which allows bicistronic expression of the GFP with the targeted gene.

Furthermore, since homologous recombination (HR) targeting efficiency in human iPS cells is low, we used a Cas9 double nickase (Cas9n) method to introduce a double-strand break (DSB) in DNA to facilitate HR and thus improve the targeting efficiency^24^,^25^. Our data confirms the efficiency of HR targeting using this approach and we have validated proper in-frame targeting using sequencing. Finally to prove the functionality of the reporter cassette, we have used artificial transcriptional activation using a dead Cas9-VP160 (dCas9 activator) approach as well as embryoid body differentiation to sort and enrich the MYF5-GFP^+^ myogenic cells^26^,^27^. This research validates the generation of knock-in myogenic reporters using the Cas9 system in human iPS cells which can be used for *in vitro* myogenic differentiation of human iPS cells.

## Results

### Cas9n pairs can target the MYF5 locus in 293T cells

**Figure 1:**
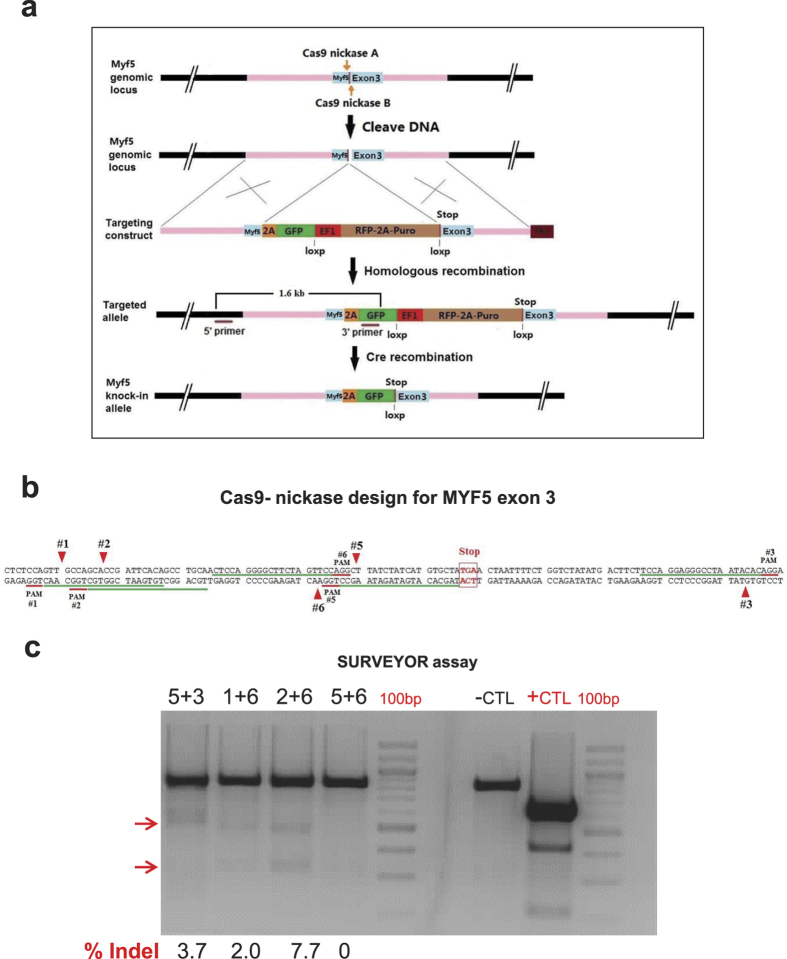
MYF5 gene targeting strategy and sgRNA design and evaluation. (**a**)The image describes the reporter knock-in strategy. After introduction of a DSB at the MYF5 exon 3 near the stop codon, a bicistronic 2A-GFP reporter cassette along with an excisable selection marker (EF1a-RFP-2A-Puro) is inserted using a homologous recombination (HR) vector. Finally after selection of the targeted vector and confirmation by sequencing, the selection cassette is excised by transient Cre expression. (**b**) The MYF5 gene was targeted at the last exon (exon 3) before the stop codon and flanking regions were analyzed for potential sgRNA target regions. Five sgRNAs along with their nicking sites are marked as well as their associated NGG PAM sequences. (**c**) The gel demonstrates cutting efficiency of each pair in 293T cells using SURVEYOR assay including the indel % quantification at bottom. Red arrows indicate approximate expected bands. Pairs 6 + 2 and 5 + 3 were chosen for subsequent experiments.

In order to design the sgRNAs for Cas9n pairs, the human MYF5 gene was analyzed to identify suitable target sites. Figure 1a describes our targeting strategy for generating a MYF5-2A GFP reporter using Cas9n mediated homologous recombination. The MYF5 gene belongs to a family of transcription factors known as myogenic regulatory factors (MRFs) which are important for cell entry and specification into skeletal myogenic program^21^,^23^,^28^. MYF5 is the first myogenic gene which starts to express in dermomyotome and it contains 3 exons encoding a single transcript.

To generate the Cas9 mediated knock-in reporter lines for MYF5 in human iPS cells, first we analyzed the 100 bp regions flanking the stop codon in the MYF5 exon 3. As shown in Fig. 1b, 4 pairs of potential sgRNA target sites were identified and annealed oligos were cloned into Cas9n vectors to generate each pair. Each pair was then transfected into 293T cells and a SURVEYOR assay was performed to determine cutting efficiency for each pair. These data are shown in Fig. 1c. As demonstrated, tested pairs demonstrated expected cutting bands after digestion with a SURVEYOR nuclease indicating their cutting ability for the targeted region. Based on % indel efficiency, pairs 2 + 6 and 5 + 3 were chosen for subsequent experiments. Later on, while targeting iPS cells, pair 5+3 demonstrated better targeting efficiency in human iPS cells.

### Targeting vector construction using recombineering

Next we made the targeting vector for MYF5. As described in Supplementary Figure 1, the targeting locus along with both homology arms were captured using recombineering (**a**) and a 2A-GFP reporter along with the selection cassette was inserted using a blunt-end ligation (**b**). Later on, the backbone vector was switched into a gateway vector which contained the negative selection (*tk*) along with unique linearization sites (**c**). The final vector was validated by sequencing to ensure in-frame positioning of the reporter/selection cassette.

### Generation of human iPS reporter clones for MYF5

After confirmation of the Cas9n pairs and targeting vector, human iPS cells were used to generate reporter lines for the MYF5. For this reason, a well characterized integration-free human iPS cell line (DF19-9 human iPS cell line generated by Dr. James Thompson’s lab obtained from WiCell) was chosen for generation of the reporter clones^29^.

**Figure 2:**
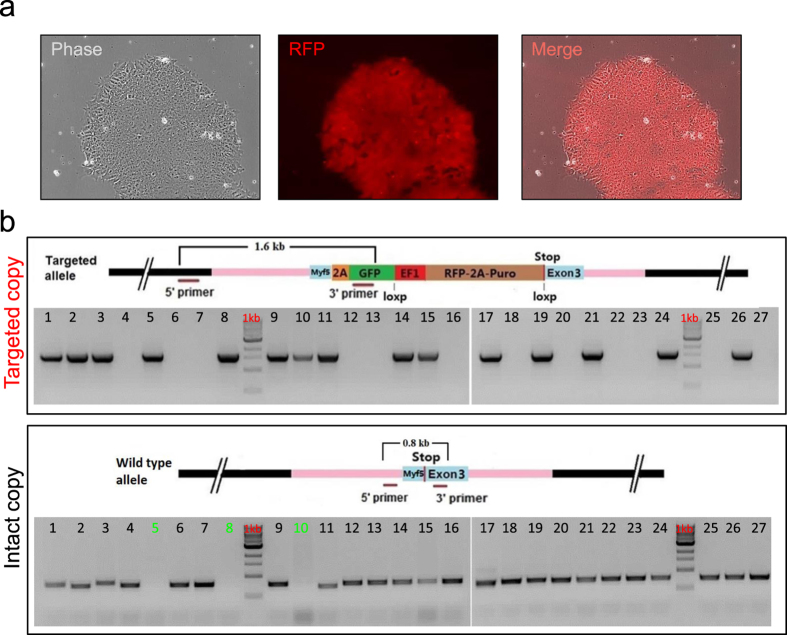
Generation of knock-in human iPS cell lines for MYF5. (**a**) The image demonstrates a single cell derived targeted human iPS clone 2 weeks after selection with uniform expression of RFP. (**b**) The gel demonstrates the PCR strategy to identify homozygous clones for the targeting vector. Upper gel indicates 15 out of 27 clones have at least one copy of targeted allele (targeting efficiency of 55%). Lower gel shows wild type band. As shown, 3 clones (marked in green numbers) out of 15 positive clones lacked the wild type band indicating both alleles are targeted (20% of targeted clones).

Human iPS cells were expanded in feeder-free Matrigel coated plates in conditioned medium and after harvest, electroporated with targeting vector cocktails containing Cas9n pairs and the HR targeting vector for the MYF5. The cells were plated after electroporation and positive and negative drug selection was started three days later for the duration of two weeks. During this time, individual resistant targeted iPS clones started to grow and expressed RFP as demonstrated in Fig. 2a. RFP-positive clones were then picked up and screened for further confirmation.

In order to test in-frame positioning of the reporter cassette, we have used a PCR screening method for the transgene. As shown in Fig. 2b (upper gel image), PCR screening confirmed the proper targeting of selected clones as 15 out of 27 clones had at least one copy of the targeted allele (55% efficiency to target at least one copy).

Positive clones then were evaluated for homozygous cloning of the reporter cassette using a PCR screen. Interestingly, as shown in Fig. 2b (lower gel image), 3 out of 15 positive clones (marked by green numbers) lacked the wild-type band indicating homozygous targeting for the MYF5 reporter cassette. Therefore, homozygous clones were selected for further characterization and differentiation studies. This is important for the reporter activity evaluation since 2A-GFP allows for bicistronic expression of target gene along with GFP. Therefore, if both copies include the reporter cassette, the GFP level would be brighter compared to the one copy targeted clones.

### Reporter clones show in-frame positioning and normal pluripotency markers

**Figure 3:**
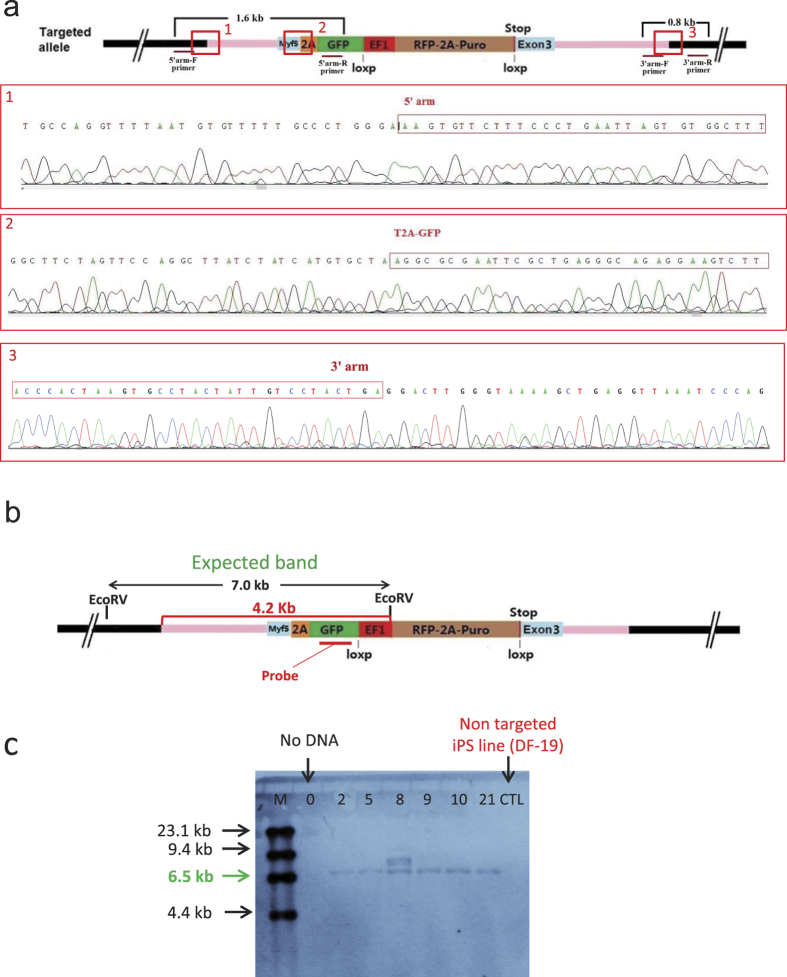
Sequencing and southern blot validation of MYF5-GFP reporter iPS clones. (**a**) Box 1 and 2 sequencing results of the 1.6 kb PCR product (from 5′ side) in one of the positive clones show in- frame positioning of the MYF5 reporter cassette. Box 1 covers the junction of 5′ homology arm with genomic DNA and box 2 covers the junction of exon 3 with the reporter cassette. No indels were detected in the represented clone. Furthermore, box 3 sequencing of the 0.8 kb PCR product of 3′ end also proves in-frame positioning of the cassette without any indels. (**b**) The image describes a southern blot strategy for confirming the correct integration site of the reporter and to exclude any random integration using an internal probe for GFP region of the reporter cassette. The diagram shows expected 7 Kb band following EcoRV digestion of the genomic DNA as marked. The red bracket indicates the minimal size of the hybridized DNA/probe in case of random integration (>4.2 Kb). (**c**) After EcoRV digestion of the genomic DNA and probe hybridization, 5 out of 6 clones showed the expected single integration band of 7 kb. Only one clone (clone 8) had two integration sites which was excluded from study.

In order to confirm in-frame positioning of the reporter cassette and exclude any possible indels at the recombination sites, the 1.6 kb PCR product from the outside 5′ homology arm up to the 2A-GFP region (as shown in Fig. 3a) was sequenced.

Then two regions were analyzed to confirm the in-frame positioning of the reporter cassette and the absence of any indels (the 1^st^ site is from the genomic DNA to the 5′ homology arm junction and the 2^nd^ site is from exon 3 to the 2A-GFP junction marked by red boxes 1 and 2 in Fig. 3a).

In addition, the 3′ homology arm junction region to the genomic DNA was also amplified by PCR and the 0.8 kb product was sequenced to confirm the absence of any indel in the 3′ end. This data is presented in box 3 in the lower panel of Fig. 3a.

At last, in order to confirm the correct integration site of the transgene in the targeted clones and to exclude the clones with random integration, a southern blot assay was performed using an internal probe for the GFP region. This data is demonstrated in Fig. 3b and c. As indicated, among the six screened clones, we were able to identify five clones with a correct single integration site (7 Kb band). Later on, the selection cassette was removed by transient Cre expression^30^ and confirmed by PCR and direct fluorescent imaging confirming the lack of RFP (Supplementary Figure 2).

Finally, the selected reporter iPS clones were evaluated for pluripotency markers including Oct3/4, Sox2, Nanog, Lin28 and SSEA-4 to confirm their cell stemness after targeting and selection experiments. These data indicated reporter clones still keep their pluripotency markers (Supplementary Figure 3). After completion of all above mentioned experiments, the clone 5 (a homozygous clone for reporter cassette) was chosen for characterization and differentiation experiments below.

### Reporter activity validation using dCas9 activator

**Figure 4:**
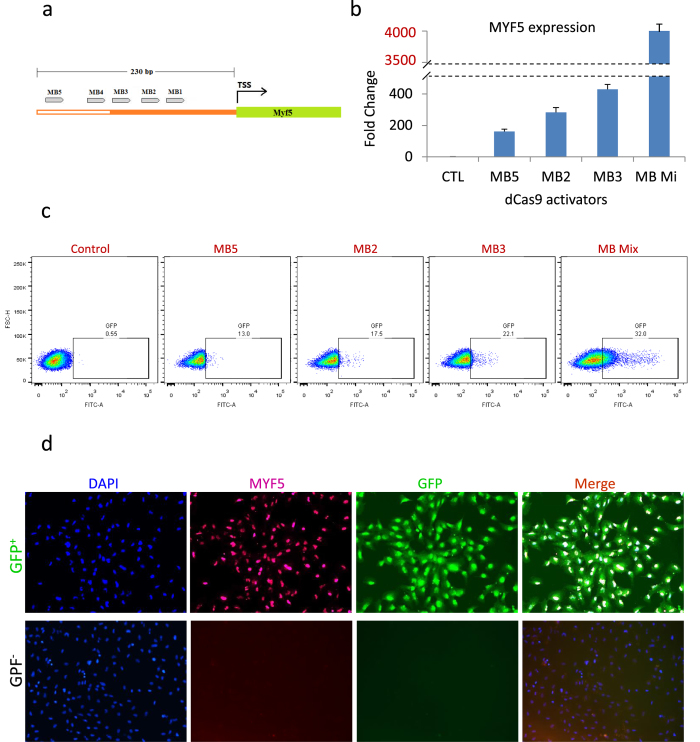
dCas9-VP160 mediated activation of MYF5 gene proves proper reporter activity in targeted clones. **(a)** The diagram shows an open chromatin region (in orange) upstream of human MYF5 gene used for sgRNA design which includes a 230 bp sequence identified by DNase I hypersensitivity clusters data in UCSC genome browser database. Five sgRNAs were selected for induction. The sequence of each sgRNA and their oligos are listed in Supplementary Figure 4. (**b**) Real time PCR for MYF5 shows its induction three days after transfection of human iPS cells with each activator or as pooled (MB Mix). The data are mean of 3 replicates and are normalized to fold of GAPDH. Among 5 tested individual activators, 3 worked fine for MYF5 induction as indicated (MB2, 3, 5). As expected pooled transfection (MB Mix) demonstrated maximal MYF5 induction. (**c**) FACS profile of non-induced vs. dCas9 activator induced reporter iPS cell clone indicates proper GFP reporter activity 3 days after transfection of the reporter iPS cells with dCas9- MYF5 activators. Individual and pooled activation GFP FACS profile is demonstrated. After pooled transfection, 32% of the cells expressed GFP reporter for MYF5. (**d**) Immunostaining on the dCas9 MYF5 activated (GFP^+^) sorted cells from a MYF5-GFP reporter iPS clone confirms proper co-expression of MYF5 along with GFP reporter and validates the functionality of the reporter iPS cells. As demonstrated in lower panel, sorted GFP negative cells did not show any MYF5 or GFP expression.

After generation of reporter human iPS cell clones and confirmation of in-frame positioning of the reporter cassette and copy number, a validated homozygous reporter human iPS lines for MYF5 (clone # 5) was tested for the reporter activity. For proof of principle, the cells were tested using a deadCas9 (dCas9-VP160) activator approach to activate the endogenous MYF5 gene^31^. Therefore, as the Fig. 4a demonstrates, the sgRNA target regions upstream to transcription start site (TSS) for MYF5 were analyzed.

In order to do this, after identification of the open chromatin region for the MYF5 gene using DNase I hypersensitivity data (Supplementary Figure 4), target sgRNA sites were determined and annealed oligos were cloned into a dCas9-VP160 vector. For MYF5 activation, five individual sgRNA activator vectors were designed and constructed. (The sgRNA sequences and their oligos are demonstrated in Supplementary Figure 4). Then each activator (individually or as pooled) was transfected into reporter iPS clones and two days later, the RNAs were harvested for RT-PCR for the MYF5 gene expression using a Taqman assay. As data in Fig. 4b indicates, we were able to identify three activators (MB2, 3, 5) based on the MYF5 gene expression.

Based on RT-PCR results, the cells were transfected again for MYF5-GFP activation in reporter iPS clones using confirmed activators (alone or as a pool). As demonstrated in Fig. 4c, three days after transfection, FACS analysis proved proper activation of the GFP reporter cassette by expression of the GFP in dCas9 activator transfected reporter cells (individually or as a pool) compared to non-transfected control ones. As expected, the pooled group (MB Mix) demonstrated maximum GFP induction.

Moreover, the sorted GFP positive and negative cells (from MB Mix activation) were fixed and stained for MYF5 and evaluated for co-expression of GFP and MYF5. As data on Fig. 4d indicates, while GFP negative sorted cells do not show any MYF5 or GFP expression, GFP positive (dCas9 activated) reporter cells co-express MYF5 along with GFP which confirms the proper activity of the reporter cassette following activation of endogenous MYF5 gene expression.

### Confirmation of MYF5 reporter activity during embryoid body (EB) differentiation

Finally, in order to prove the MYF5 reporter activity during *in vitro* differentiation, the human iPS MYF5-GFP reporter clones were differentiated using an EB differentiation method. In order to activate the myogenic differentiation, the EBs were treated with a Wnt agonist (CHIR99021, Stemcell Technologies Inc) for four days and analyzed for GFP activity as well as the MYF5 expression during an EB differentiation time-course.

**Figure 5:**
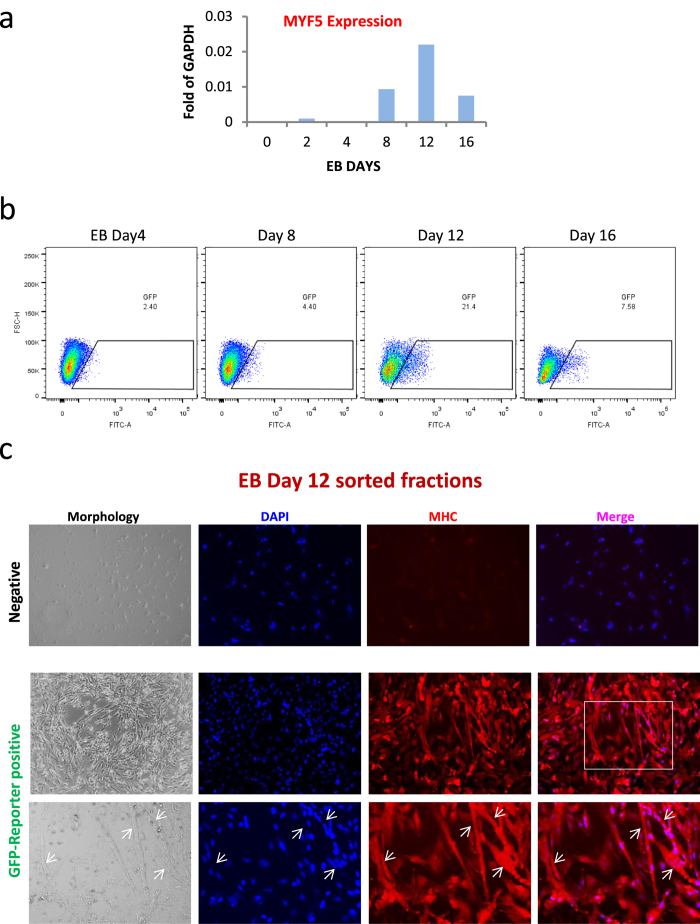
Embryoid body (EB) differentiation of a MYF5 human iPS reporter clone demonstrates time-course expression of the MYF5. (**a**) Gene expression profile of MYF5 during EB differentiation. Data are mean of 2 sets of experiments. As demonstrated, MYF5 expression starts to increase after day 8 of differentiation and reaches to maximum at day 12. (**b**) FACS profile of MYF5-GFP reporter clone during EB differentiation time-course. As demonstrated, MYF5 GFP reporter activity shows similar pattern of expression with MYF5 gene expression with maximal expression at day 12 of EB differentiation. (**c**) The morphology and immunostaining of sorted cells after five days of differentiation in myotube induction medium. For this experiments, EB day 12 was used for sort as demonstrated in left FACS panel. Positive and negative cells were sorted and plated in similar densities (10,000/cm^2^) on Matrigel-coated plates supplemented with EB medium with b-FGF. After 5 days of induction and 90% confluency, the medium switched to myotube induction condition (2% horse serum in DMEM). As shown in brightfield images on the left, 5 days after myotube induction, while majority of reporter negative cells detached and failed to undergo terminal myotube differentiation, MYF5-GFP positive reporter cells differentiated into myotubes. Immunostaining for myosin heavy chain (MHC in red) confirmed the enrichment for myogenic cells in MYF5-GFP positive fraction as demonstrated by presence of myotubes in explant. Lower panels demonstrate a higher magnification image (selected area in white box) containing multinucleated myotubes marked by arrows.

As gene expression data in Fig. 5a demonstrates, MYF5 expression started to increase around day 8 of differentiation with maximal expression at day 12. This pattern matched with the GFP reporter activity as shown in Fig. 5b by time-course FACS analysis with maximal GFP expression at EB day 12.

Furthermore, to confirm the myogenic activity of the cells, positive and negative cells from EB day 12 were sorted and expanded in EB medium on Matrigel-coated plates for a few days and were tested for myotube differentiation by switching medium to low-nutrient myotube induction medium (2% horse serum in DMEM). These data is demonstrated at Fig. 5c. As brightfield images indicate while most of the GFP negative cells detached and could not form myotubes, the MYF5-GFP positive fraction differentiated into multinucleated myotubes expressing myosin heavy chain (MHC) as demonstrated by immunostaining of the explants in Fig. 5c. These results confirmed the proper reporter activity of the MYF5-GFP reporter during *in vitro* differentiation of human iPS reporter cells.

## Discussion

Directed differentiation of the pluripotent stem cells toward desired tissue progenitors is one of the important goals in the stem cell biology. Especially with the availability of iPS cells, derivation of the tissue specific progenitors provides an excellent opportunity for *in vitro* disease modeling, drug screening, gene correction studies and eventually regenerative purposes^7^,^10^,^11^. In this regard, efforts have been done to derive skeletal myogenic progenitors from ES/iPS cells using different methods such as myogenic gene over-expression or small molecule-mediated differentiation of the cells toward myogenic progenitors^12^,^13^,^14^,^15^,^16^,^17^,^18^,^19^,^20^. However, these experiments were limited to either forced over-expression (which does not mimic normal developmental pattern) or in the case of small molecules, a retrospective evaluation of myogenic induction in the explants. Therefore, the current study was designed to develop and characterize a novel MYF5 reporter human iPS cell line which allows for prospective identification of myogenic cells.

By using a CRISPR/Cas9 double nickase mediated homologous recombination (HR), we have demonstrated a highly efficient approach for generation of the knock-in reporters in human iPS cell lines. Although other site-specific endonuclease systems such as ZFNs or TALENs are also available, Cas9-nickase system has a clear advantage due to its easy design and construction and its reduced risk of off-target side-effects^24^,^25^,^32^,^33^,^34^,^35^,^36^. As our results indicated, we have achieved a targeting rate of 55% in selected clones which demonstrates a high efficiency of Cas9-nickase mediated HR in human iPS cells.

Another aspect of this study is the successful application of Cas9-mediated gene activation for MYF5 induction in the reporter clones^26^,^27^. By design and assembly of multiple dCas9 activators for the MYF5, they have been screened for MYF5 gene activation. As results indicated, MYF5 reporter clones have been validated for proper GFP reporter activity using this method. Obviously, in this case, the aim was validation of reporter activity but this approach can also be used for directed differentiation of the human iPS cells toward myogenic lineage.

Finally in order to prove proper reporter activity in an *in vitro* differentiation setting, we have demonstrated a time-course EB differentiation approach for the evaluation of myogenic induction using gene expression and FACS analysis/sort. As indicated in the results, by using a Wnt agonist (CHIR99021) to induce myogenesis in EBs, the MYF5 gene expression as well as the GFP reporter activity can be easily identified using a gene expression assay and flow cytometry. Furthermore, sorting for MYF5-GFP positive fraction confirmed their myogenic potential as demonstrated by their *in vitro* differentiation into multinucleated myotubes.

Taken together, this study demonstrates successful generation of a knock-in MYF5-GFP reporter in human iPS cell line using a Cas9n mediated HR targeting with high efficiency. The generated reporter lines can be used for evaluation of directed differentiation of human iPS cells toward myogenic lineage using chemical screening as well as to study temporal emergence of myogenic progenitors during *in vitro* differentiation.

## Methods

### sgRNA design and Cas9 nickase (Cas9n) vector assembly

Cas9 target sites were identified using the online CRISPR design tool (crispr.mit.edu)^25^. Briefly, 200 bp DNA sequences of the human MYF5 gene flanking the stop codon (100 bp before and after the stop codon) were used for designing the sgRNAs. Later on, 4 pairs of sgRNAs were selected to result in an optimal 5´-overhang arm length to ensure efficient cutting^24^. Then for each target site, a specific Cas9n vector was made. Briefly, the Cas9 nickase vector (hSpCas9n nickase- pX335, # 42335 from addgene)^37^ was digested using BbsI and a pair of annealed oligos (20 bp target sequences) were cloned into the guide RNA locus^25^. Finally, vectors were sequenced to ensure the presence of the right sequence.

### SURVEYOR assay for Cas9n pairs

Assembled Cas9n pairs were tested on 293T cells using transfection. Briefly, 0.5 μg of each plasmid was used for transfection of 293T cells. Three days after the transfection, DNA from the targeted and non-targeted cells was harvested and PCR-amplified for the targeted region. Then, the PCR products were hybridized using a thermocycler to allow heteroduplex formation from the targeted and non-targeted cells DNA. Later on, 400 ng of hybridized DNA was treated with the SURVEYOR nuclease and DNA fragments were analyzed using agarose gel electrophoresis to identify the presence of the expected bands.

### Targeting vector design and construct

BAC clone for the human MYF5 was obtained from CHORI (BAC RP11-626E8). For construction of the targeting vector, we used a highly efficient recombination based cloning method in bacteria which was developed by Dr.Capecchi’s group^38^. Briefly, the homology arms (5′ arm:1.3 kb; 3′ arm: 6 kb) along with the target region were captured into a gateway compatible vector (pStart-K, # 20346 from addgene)^38^ using recombineering. After the introduction of a cutting site with a selection sequence (AscI-cat-AscI) using another recombineering reaction, the reporter with an excisable selection sequence (2A-GFP-loxP-EF1a-RFP-2A-Puro-loxP fragment from HR130PA-1 vector, System Biosciences-SBI) was cloned into the AscI cutting sites using a blunt-end ligation. Finally, through a gateway recombination, the modified genomic fragment was switched into another vector which contained the negative selection (*tk*) as well as unique linearization sites. The final vector was linearized before its use for targeting.

### Generation of the MYF5 reporter human iPS clones and selection

After the validation of Cas9n pairs using the SURVEYOR assay, a fully characterized integration-free human iPS cell line (DF19-9, WiCell)^29^ was used for generation of human reporter clones. The human iPS cells were expanded on Matrigel in feeder-conditioned medium for a few days and after harvesting, 1 × 10^7^ cells were electroporated (Gene Pulser Xcell -Bio-Rad, 250 V, 250 μF, ∞ Ώ) using 10 μg of Cas9 vectors plus 50 μg of the linearized targeting vector for MYF5. After the electroporation, cells were expanded for a few days and selection was done by adding positive and negative selection (0.75 μg/ml Puromycin and 200 nM FIAU) for 2 weeks. After the selection, RFP positive clones were picked up for expansion and PCR screening.

### PCR and sequencing validation of reporter clones

PCR primers were designed to span the outside 5′ homology arm up to 2A-GFP region to ensure proper site-specific targeting. This region (1.6 kb amplicon) was PCR-amplified and sequenced to ensure in-frame positioning of the reporter/selection fragment and to rule out the presence of indels at recombination site. 3′ end was also amplified using PCR (0.8 kb amplicon) and sequenced for analysis. Furthermore, to identify the clones with both allele (homozygous) reporters, PCR primers were designed to identify intact MYF5 as well as the targeted allele.

### Southern blotting validation of transgene copy number

In order to prove single integration of the transgene, a southern blot strategy was designed using an internal probe within the GFP region. The genomic DNA from the Myf5 reporter hiPS cells was extracted and verified by PCR. 5 μg of genomic DNA from each cell line was digested with EcoRV, separated in a 0.9% agarose gel and transferred to a Hybond N^+^ membrane as described in details before^39^. The genomic DNA on the membrane was then hybridized with a Digoxigenin (DIG)-labeled internal probe derived from partial GFP sequence as demonstrated in Fig. 3b.

### Cre removal of the selection cassette

After characterization of homozygous clones, the selection cassette was removed by transient expression of a Cre plasmid (pCAG-Cre-IRES2-GFP Addgene # 26646) and was validated using PCR screening and direct fluorescent imaging.

### sgRNA design for dCas9-VP160 activator and vector assembly

In order to evaluate reporter activity in targeted iPS clones, we have used a nuclease-dead Cas9 activator system (pAC154-dual-dCas9VP160-sgExpression # 48240 from addgene)^31^. The sgRNAs were designed to target 230 bp upstream of MYF5 based on DNase I hypersensitivity clusters available at UCSC genome browser database. Multiple target sites were identified and annealed oligos were cloned into the activator vector. Generated vectors were sequenced to ensure the presence of target sequence.

### Reporter validation using dCas9-VP160 transient activation

dCas9-VP160 activator constructs were tested on human iPS cells to validate their efficiency for gene activation. Individual or pooled constructs (1 μg construct for individual, 0.5 μg of each for mix, 4–5 × 10^5^ cells/well of 24 well plate with 70–90% confluency on the day of transfection) were transfected into human iPS cells using Lipofectamine LTX transfection reagent (Life technologies, 2.5 μg/each dCas9 activator plasmid and 7.5 μl Lipofectamine LTX for each well of 6-well plate). Three days after the transfection, cells were harvested for RNA extraction. cDNA was made using reverse-transcription and RT-PCR was performed using Taqman assays for human MYF5 to identify the best activators. After choosing the best activators, MYF5 -GFP knock-in human iPS cells were transfected again and were harvested for GFP analysis using a FACS Aria system and were gated against non-induced cells. For immunostaining, sorted cells were sorted and fixed using 4% PFA/PBS and after blocking, were stained for MYF5 (1:100, SC-302 from Santa Cruz Biotechnology), GFP (1:250, ab13970 from Abcam) and DAPI. For secondary antibodies, we have used the appropriate Alexa Fluor antibodies (1:500, 488 goat anti-chicken and 555 goat anti-rabbit from Life Technologies). For myotube staining, fixed cells were stained for myosin heavy chain-MHC (1:20, MF-20 from hybridoma bank) overnight in 4 degree and later counterstained with appropriate secondary antibody.

### iPS cell expansion and embryoid body (EB) differentiation

Human iPS cells were cultured in E8 essential medium (Life Technologies) and expanded according to its protocol. For EB generation, cells were harvested using EDTA and EBs were formed on a shaker as described before^12^. EB medium consisted of IMDM supplemented with 10% of horse serum and 20% of fetal bovine serum plus penicillin/streptomycin. For myogenic induction, the EBs were induced by a Wnt agonist (3 μM of CHIR99021, Stem Cell Technologies) during the first 4 days and then expanded by basic FGF alone (10 ng/ml). EB day 12 sorted cells were also expanded in EB medium on Matrigel coated plates for a week and were differentiated into myotubes using myotube induction medium (2% horse serum in low glucose DMEM).

## Additional Information

**How to cite this article**: Wu, J. *et al.* Generation and Characterization of a MYF5 Reporter Human iPS Cell Line Using CRISPR/Cas9 Mediated Homologous Recombination. *Sci. Rep.* **6**, 18759; doi: 10.1038/srep18759 (2016).

## Supplementary Material

Supplementary Information
