# Supplementary material for: Generation and Characterization of a MYF5 Reporter Human iPS Cell Line Using CRISPR/Cas9 Mediated Homologous Recombination
Source: Sci Rep. 2016 Jan 5;6:18759. doi: 10.1038/srep18759 (PMC4700424; doi:10.1038/srep18759)
Supplement: Supplementary Information [file srep18759-s1.pdf]

Supplementary information

Title:

Generation and characterization of a MYF5 Reporter Human iPS Cell Line Using CRISPR/Cas9 Mediated Homologous Recombination

Jianbo Wu[1], Samuel D. Hunt[1], Haipeng Xue[1, 2], Ying Liu[1, 2, 3], Radbod Darabi[1]*

[1]Center for Stem Cell and Regenerative Medicine (CSCRM),
[2]Department of Neurosurgery
[3]The Senator Lloyd & B.A. Bentsen Center for Stroke Research
The Brown Foundation Institute of Molecular Medicine (IMM)
University of Texas Health Science Center at Houston
Houston, TX 77030, USA

***Corresponding author:**

Radbod Darabi, M.D., Ph.D.
Assistant Professor
Center for Stem Cell and Regenerative Medicine/ Brown Foundation Institute of Molecular Medicine
University of Texas Health Science Center at Houston
1825 Pressler Street, SRB 630A
Houston, TX 77030
Phone: (713) 500-3411
Fax: (713) 500-2424
Email: radbod.darabi@uth.tmc.edu

Key words: MYF5 reporter, human iPS cells, skeletal muscle, Cas9, CRISPR, dCas9, Cas9n

Supplementary figures:

Supplementary Figure 1- Targeting vector design and construction

The image describes targeting vector construction using recombineering. As shown, first: (**a**) genomic fragment containing targeting exon and homology arms was captured in a gateway vector using recombineering in bacteria. Subsequently rare AscI cutting sites were introduced before the stop codon for (**b**) cut and paste in-frame cloning of 2A-GFP along with selection markers. (**c**) Finally the vector was switched to another Gateway vector containing negative selection and linearization site.

Supplementary Figure 2- Cre removal of selection cassette in MYF5 reporter human iPS clones

a) PCR strategy for identification of clones without selection cassette after transient Cre expression.

b) PCR gels show the presence of 1.6 kb targeting band and absence of 1.1 kb selection band confirming proper Cre removal of the selection cassette in different clones.

c) Direct fluorescence imaging confirms the Cre removal of the selection cassette and the absence of RFP.

Supplementary Figure 3- Targeted human iPS cells retain their pluripotency markers

a) The targeted human iPS cells show normal morphology and uniformly express SSEA-4.

b) The immunostaining for pluripotency markers demonstrates uniform expression of these markers.

Supplementary Figure 4- sgRNA design for dCas9-VP160 MYF5 activation

a) A 230 bp upstream region of MYF5 was used for sgRNA design based on its open chromatin DNase I hypersensitivity data.

b) The table shows individual sgRNA sequences and their synthesized oligos. Among tested activators, MB1 and MB4 did not show any MYF5 activation and were excluded later.

Supplementary Figure-1

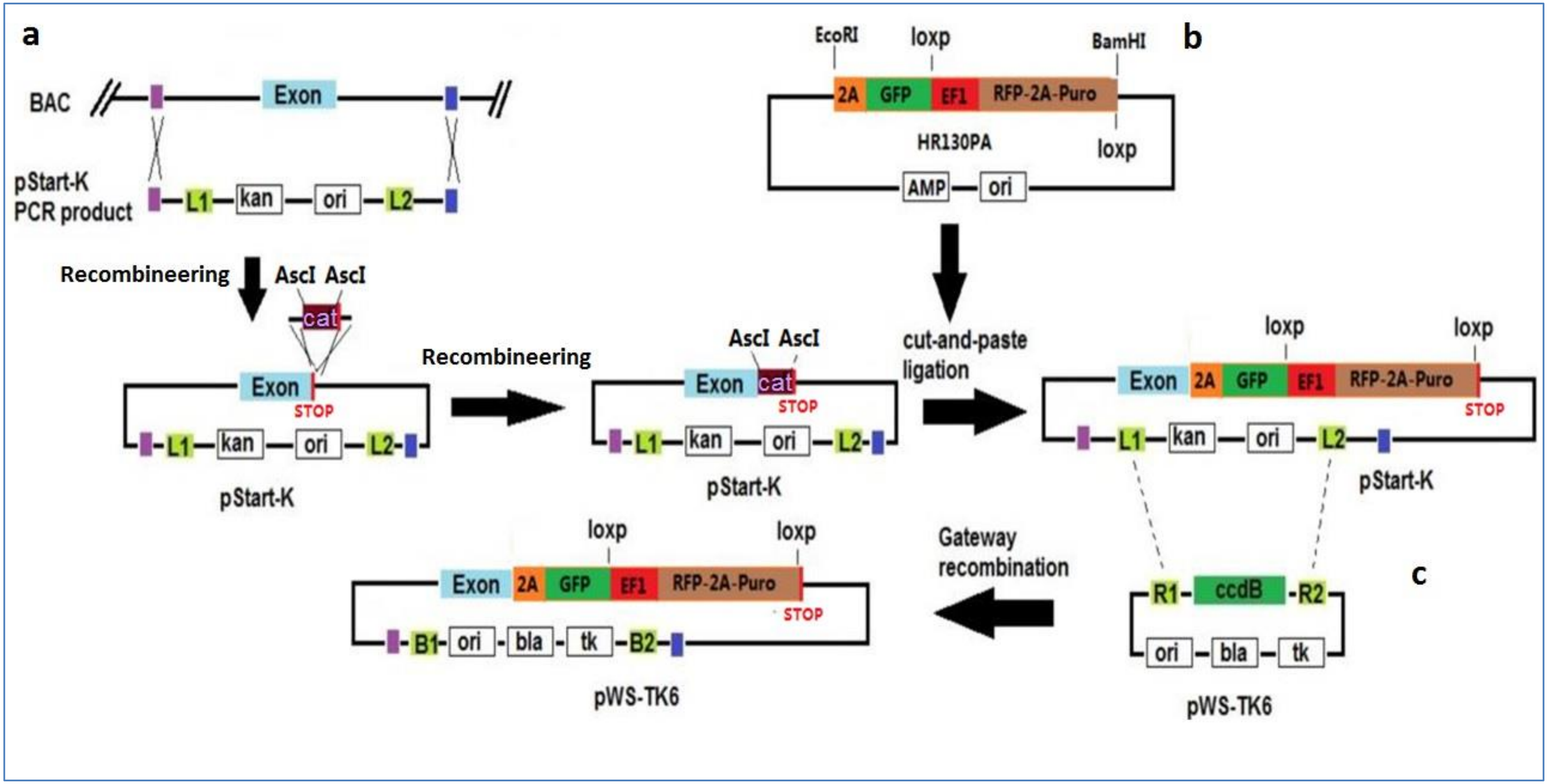

Supplementary Figure-2

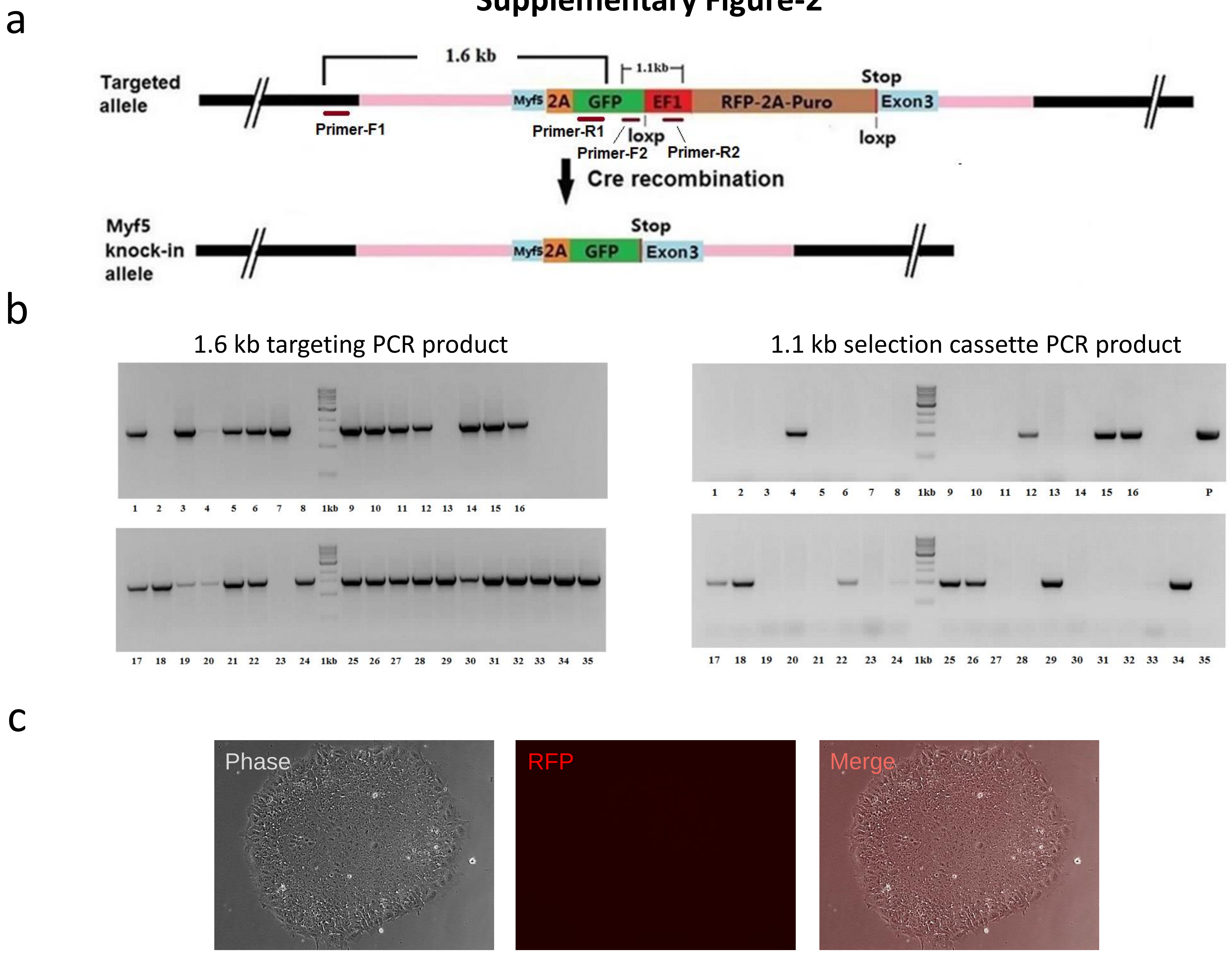

Supplementary Figure-3

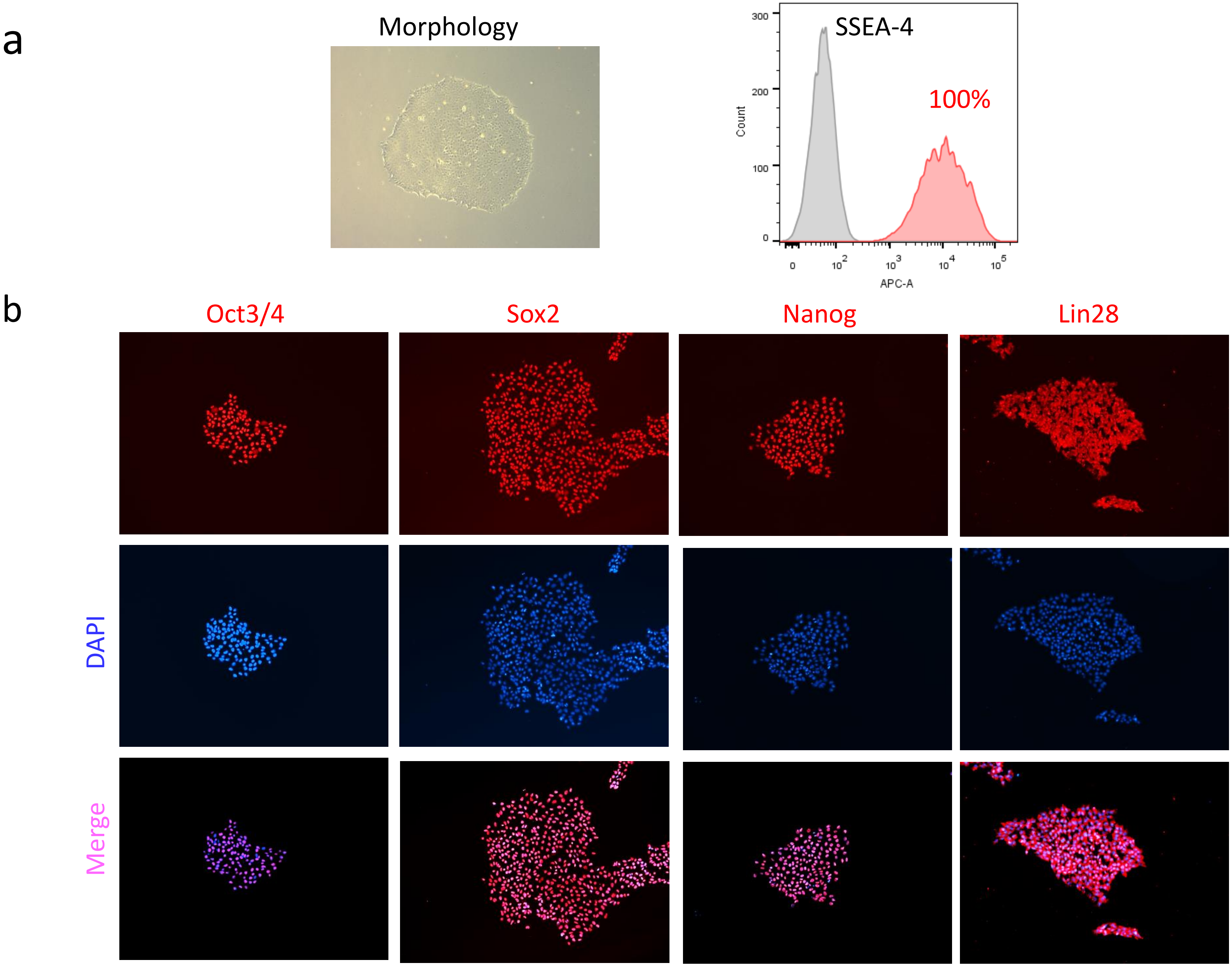

Supplementary Figure-4

MYF5 Upstream Sequence Used for dCas9 Design

AGAGAGTGCAAATGAGCAGGTTGGGGACCCACTGCCGCCCCAGCAGAAATACAGTAAAACCATTAGCGTCAAAAGGGGCAGTACACAGCAGATTGTCTGTAG
AGGAAAGGCGGAGGTTTGTCCAGACAGCCCCCGCGGGGTTGCGGTGGGATATGCTAATAGTGCCTGGCCACTGGGGCCGGCCTCCCTCCCGAAAGAATATA
TAAAGAACCCCAACCCCAGCTTGTCT

sgRNAs used for dCas9- VP160 mediated MYF5 activation

Name	Target sequence	Forward Oligo	Reverse Oligo
MB1	TGGGATATGCTAATAGTGCCTG G	caccgTGGGATATGCTAATAGTGC C	aaacGGCACTATTAGCATATCCCAc
MB2	TTGTCCAGACAGCCCCCGCGGG G	caccgTTGTCCAGACAGCCCCCG CG	aaacCGCGGGGGCTGTCTGGACA Ac
MB3	ACACAGCAGATTGTCTGTAGAG G	caccgACACAGCAGATTGTCTGTA G	aaacCTACAGACAATCTGCTGTGT c
MB4	GTAAAACCATTAGCGTCAAA AGG	caccGTAAAACCATTAGCGTCAA A	aaacTTTGACGCTAATGGTTTTAC
MB5	GAGTGCAAATGAGCAGGTTGG GG	caccGAGTGCAAATGAGCAGGTT G	aaacCAACCTGCTCATTTGCACTC
